# The SARS-CoV-2 Pandemic at the Wildlife–Domestic Animal–Human Interface

**DOI:** 10.3390/pathogens12020222

**Published:** 2023-01-31

**Authors:** Giovanni Di Guardo

**Affiliations:** General Pathology and Veterinary Pathophysiology, Veterinary Medical Faculty, University of Teramo, Località Piano d’Accio, 64100 Teramo, Italy; gdiguardo@unite.it; Tel.: +39-347-6317862

Since the start of the COVID-19 pandemic, which has hitherto killed almost 7 million people worldwide—although the true mortality figures could be much higher—we have witnessed a progressively expanding number of domestic and wild mammalian species acquiring Severe Acute Respiratory Syndrome CoronaVirus-2 (SARS-CoV-2) infection, both spontaneously and experimentally [1].

The progressively expanding SARS-CoV-2 host range, hitherto encompassing more than thirty wildlife and domestic species, could be plausibly linked, among others, to the development of new, highly contagious, and/or pathogenic variants of concern (VOCs) and variants of interest (VOIs) of this pandemic betacoronavirus.

Over the past three years, in fact, a huge number of mutational events were recorded in the genetic make-up of SARS-CoV-2, with this leading to the global appearance of several VOCs and VOIs (such as those termed “alfa”, “beta”, “gamma”, “delta”, and the highly contagious and immune-evasive “omicron”, alongside its BA.1-BA.5 subvariants and the more recently identified ones named “Centaurus”, “Chiron”, “Gryphon”, and “Cerberus”, followed by the newly emerged and highly transmissible “Kraken”). The progressive acquirement of “non-silent” mutations in the SARS-CoV-2 genome is directly connected to an enhanced viral replication and, provided that the virus genetic make-up consists of approximately 30,000 bases, each replication cycle will imply as an average the occurrence of 1 mutation/10,000 nucleotides [2]. Indeed, by progressively undergoing mutational events in both naturally and “artificially” gregarious species such as white-tailed deer and mink, respectively, the possibility that new, highly divergent, and pathogenic SARS-CoV-2 lineages could emerge from “animal communities” and infect people should be seriously considered.

Although the vast majority of SARS-CoV-2 VOCs and VOIs have developed in humans, some of them have also happened to “spill back” from animals to mankind. This is clearly shown, for example, by the recent case of a human infection caused by a highly divergent SARS-CoV-2 lineage (B.1.641) circulating among white-tailed deer (*Odocoileus virginianus*) from the Canadian region of Ontario, harboring 76 mutations (37 of which had not been previously detected in human viral isolates) and sharing a quite recent common ancestry with a mink SARS-CoV-2 strain from Michigan [3]. Indeed, white-tailed deer have already been shown to be particularly susceptible to SARS-CoV-2 infection on the basis of a high homology degree of their angiotensin-converting enzyme-2 (ACE-2) viral receptor with the human one, thereby supporting in a very efficient way the intraspecies transmission of several VOCs and VOIs infecting people [4,5]. Furthermore, a vast proportion (40%) of white-tailed deer from North-Eastern USA were proven to harbor anti-SARS-CoV-2 antibodies in their blood serum [6], with the omicron variant having been also identified in deer from New York State and Ohio [7].

Still noteworthy, during the spring/summer seasons of 2020, the “cluster 5” VOC, characterized by the S:Y453F mutation, emerged from intensely bred mink in Denmark and the Netherlands. Following transmission from infected people (viral spillover), in fact, SARS-CoV-2 was shown to evolve into the aforementioned VOC inside the body of mink, which subsequently “returned” the mutated virus to humans (viral spillback) [8]. This led, in turn, to the “stamping-out” of 17 million minks in Denmark due to the public health hazard posed by them.

As far as concerns the transmission of SARS-CoV-2 from people to animals, cases of infection caused by the “alfa” variant were described in two cats and in one dog from France with suspected myocarditis, whose owners had shown COVID-19-associated respiratory symptoms three to six weeks before [9]. It has also been claimed that pet hamsters transferred the highly pathogenic “delta” VOC to pet shop workers and visitors in Hong Kong [10], while the omicron BA.2 subvariant could have been passed to people in China by a dog acting as a passive SARS-CoV-2 mechanical carrier [11].

Among the wild animal species hitherto deemed susceptible to SARS-CoV-2 infection, a number of them appear to be increasingly threatened by extinction in terrestrial as well as in marine ecosystems.

While being of great concern, this simultaneously provides a strong argument for advocating the opportunity, if not the need, of immunizing the aforementioned species against SARS-CoV-2, although we do not know yet “how” and “to which extent” SARS-CoV-2 infection could impact their health and conservation status. By doing so, in fact, we would correctly apply the so-called “principle of precaution”, thus aiming at protecting the increasingly threatened animal biodiversity by conferring an adequate antiviral population immunity to those SARS-CoV-2-susceptible wildlife species facing an increased extinction risk (e.g., lions, tigers, snow leopards, gorillas, etc.) [12]. At the same time, we would likely contribute to reducing SARS-CoV-2 circulation and, consequently, the appearance of new, highly transmissible and/or pathogenic VOCs.

To this aim, the tremendous progress gained in the production of the currently available vaccines through the messenger RNA (mRNA) technology should be viewed as a great advantage and scientific achievement.

Within this framework, the SARS-CoV-2 vaccination programs should also include animals either living in close contact with people or intensely bred (i.e., mink and pigs), as well as wildlife species with a marked social ecology that have been shown to enhance the intra-species transmission of SARS-CoV-2 (i.e., white-tailed deer) [5,6].

The first key lesson we have learned from the dramatic SARS-CoV-2 pandemic is that human, animal, and environmental health are mutually and inextricably linked to each other. Additionally, this is the reason why, in order to be better prepared for future pandemics, we urgently need to adopt a scientific evidence-based, “holistic”, multidisciplinary, and “One Health-based” approach.

In this respect, I conclude that it is very surprising, if not almost unbelievable, that in the far too brief two years of its life, the “Italian CoViD-19 Scientific Committee” (popularly known by the acronym “CTS”) has never appointed any veterinarians as members of the committee, thereby completely forgetting that at least 70% of the pathogens responsible for emerging infectious diseases (including also SARS-CoV-2 and its two betacoronavirus “predecessors”, SARS-CoV and MERS-CoV) have either a proven or suspect origin from one or more animal reservoirs [13].

*Errare Humanum est Perseverare Autem Diabolicum*!

## References

[1] Jemeršić L., Lojkić I., Krešić N., Keros T., Zelenika T.A., Jurinović L., Skok D., Bata I., Boras J., Habrun B. (2021). Investigating the Presence of SARS-CoV-2 in Free-Living and Captive Animals. Pathogens.

[2] Di Guardo G. (2022). Is gain of function a reliable tool for establishing SARS-CoV-2 origin?. Adv. Microbiol..

[3] Pickering B., Lung O., Maguire F., Kruczkiewicz P., Kotwa J.D., Buchanan T., Gagnier M., Guthrie J.L., Jardine C.M., Marchand-Austin A. (2022). Divergent SARS-CoV-2 variant emerges in white-tailed deer with deer-to-human transmission. Nat. Microbiol..

[4] Palmer M.V., Martins M., Falkenberg S., Buckley A., Caserta L.C., Mitchell P.K., Cassmann E.D., Rollins A., Zylich N.C., Renshaw R.W. (2021). Susceptibility of white-tailed deer (*Odocoileus virginianus*) to SARS-CoV-2. J. Virol..

[5] Hale V.L., Dennis P.M., McBride D.S., Nolting J.M., Madden C., Huey D., Ehrlich M., Grieser J., Winston J., Lombardi D. (2022). SARS-CoV-2 infection in free-ranging white-tailed deer. Nature.

[6] Chandler J.C., Bevins S.N., Ellis J.W., Linder T.J., Tell R.M., Jenkins-Moore M., Root J.J., Lenoch J.B., Robbe-Austerman S., DeLiberto T.J. (2021). SARS-CoV-2 exposure in wild white-tailed deer (*Odocoileus virginianus*). Proc. Natl. Acad. Sci. USA.

[7] Wetzel C. (2022). Discovery of Omicron in New York Deer Raises Concern over Possible New Variants. Smithson. Mag..

[8] Lassaunière R., Fonager J., Rasmussen M., Frische A., Polacek C., Rasmussen T.B., Lohse L., Belsham G.J., Underwood A., Winckelmann A.A. (2021). In vitro Characterization of Fitness and Convalescent Antibody Neutralization of SARS-CoV-2 Cluster 5 Variant Emerging in Mink at Danish Farms. Front. Microbiol..

[9] Ferasin L., Fritz M., Ferasin H., Becquart P., Corbet S., Ar Gouilh M., Legros V., Leroy E.M. (2021). Infection with SARS-CoV-2 variant B.1.1.7 detected in a group of dogs and cats with suspected myocarditis. Vet. Rec..

[10] Mesa N. (2022). Pet Hamsters Spread SARS-CoV-2 in Hong Kong: Preprint. TheScientist.

[11] Zhou C., Wu A., Ye S., Zhou Z., Zhang H., Zhao X., Wang Y., Wu H., Ruan D., Chen S. (2022). Possible transmission of COVID-19 epidemic by a dog as a passive mechanical carrier of SARS-CoV-2, Chongqing, China, 2022. J. Med. Virol..

[12] Delahay R.J., de la Fuente J., Smith G.C., Sharun K., Snary E.L., Flores Girón L., Nziza J., Fooks A.R., Brookes S.M., Lean F.Z.X. (2021). Assessing the risks of SARS-CoV-2 in wildlife. One Health Outlook.

[13] Casalone C., Di Guardo G. (2020). COVID-19 and Mad Cow Disease: So Different Yet so Similar. Science.

